# O.6.3-1 Implementation and evaluation of the Walking In ScHools (WISH) Trial in adolescent girls: mixed-methods process evaluation of a clustered randomised control trial using RE-AIM

**DOI:** 10.1093/eurpub/ckad133.281

**Published:** 2023-09-11

**Authors:** Angela Carlin, Leanne C Doherty, S Maria O'Kane, Russell Jago, Gary McDermott, Alison M Gallagher, Ian M Lahart, Maria Faulkner, Marie H Murphy

**Affiliations:** Centre for Exercise Medicine, Physical Activity and Health, Sports and Exercise Sciences Research Institute, Ulster University, Magee Campus, Derry BT48 7JL, UK; Centre for Exercise Medicine, Physical Activity and Health, Sports and Exercise Sciences Research Institute, Ulster University, Magee Campus, Derry BT48 7JL, UK; Centre for Exercise Medicine, Physical Activity and Health, Sports and Exercise Sciences Research Institute, Ulster University, Magee Campus, Derry BT48 7JL, UK; Centre for Exercise, Nutrition & Health Sciences, School for Policy Studies, University of Bristol, Bristol BS8 1TZ, UK; Centre for Exercise Medicine, Physical Activity and Health, Sports and Exercise Sciences Research Institute, Ulster University, Magee Campus, Derry BT48 7JL, UK; Nutrition Innovation Centre for Food and Health (NICHE), Biomedical Sciences Research Institute, Ulster University, Coleraine Campus, Coleraine BT52 1SA, UK; Faculty of Education, Health and Wellbeing, University of Wolverhampton, Walsall Campus, Gorway Road, Walsall WS1 3BD, UK; Department of Law and Humanities, Atlantic Technology University, Port Road, Letterkenny, Ireland; Centre for Exercise Medicine, Physical Activity and Health, Sports and Exercise Sciences Research. Institute, Ulster University, Belfast Campus, Belfast, BT15 1ED, UK; Physical Activity for Health Research Centre (PHARC), Institute for Sport, Physical Education and Health Sciences, University of Edinburgh, Edinburgh, EH8 9YL, UK; a.carlin1@ulster.ac.uk

## Abstract

**Purpose:**

The aim of the Walking In ScHools (WISH) Study was to investigate the effectiveness of a school-based, peer-led, walking intervention, delivered across the school year, at increasing adolescent girls (12-14 years) physical activity (PA) levels. The intervention was delivered in schools across Northern Ireland (n6) and the Republic of Ireland (n3). Older girls (15-18 years) were trained as Walk Leaders and led the pupils in 10–15-minute brisk walks, before school, during break and lunch recess.

**Methods:**

A mixed methods process evaluation was undertaken, using the RE-AIM framework, to evaluate the WISH intervention. Attendance and fidelity of walks were monitored by Walk Leaders completing a check list at each walk. At baseline, end of intervention and follow-up, pupils completed questionnaires to assess self-efficacy for PA and walking, health-related quality of life and reasons for engaging in PA. Friendship networks were evaluated at baseline and end of intervention. Walk Leaders completed questionnaires before the training and at end of intervention to evaluate self-perception of PA and fitness, PA enjoyment and self-efficacy, and leadership skills. Semi-structured online interviews were conducted with Teachers (n9) and Walk Leaders (n19), to identify key elements affecting implementation. Focus groups (n9) were conducted with pupils who were low and high attenders at walks, to identify factors that affected intervention participation, motivation and enjoyment. Interviews and focus groups were audio-recorded and transcribed verbatim. Qualitative data was managed in NVivo and analysis was undertaken using thematic analysis. Quantitative analysis was undertaken using SPSS (IBM SPSS V23).

**Results:**

A total of 589 pupils were recruited and 286 girls were allocated to the intervention cluster with 149 Walk Leaders trained. Over the duration of the study, 51 pupils and 4 Walk Leaders withdrew from the study. Most girls attended walks (n212; 74%), albeit, participation varied across schools, with number of walks attended, ranging from 2 to 80. Data analysis is ongoing and results will be finalised May 2023.

**Conclusions:**

Early results from the process evaluation of this fully powered Intervention, indicate implementation of the intervention varied across schools. This process evaluation will provide insights into factors affecting implementation of school-based interventions.

